# Effect of Humantenine on mRNA m6A Modification and Expression in Human Colon Cancer Cell Line HCT116

**DOI:** 10.3390/genes13050781

**Published:** 2022-04-27

**Authors:** Yajiao Wu, Xiaoying Chen, Wenqiang Bao, Xinyu Hong, Chutao Li, Jiatong Lu, Dongcheng Zhang, An Zhu

**Affiliations:** 1 Key Laboratory of Ministry of Education for Gastrointestinal Cancer, School of Basic Medical Sciences, Fujian Medical University, Fuzhou 350108, China; wuyajiao@fjmu.edu.cn; 2 Department of Pathogen Biology, School of Basic Medical Sciences, Fujian Medical University, Fuzhou 350108, China; 3 Experimental Teaching Center of Basic Medical Sciences, Fujian Medical University, Fuzhou 350108, China; xychen@fjmu.edu.cn; 4 School of Basic Medical Sciences, Fujian Medical University, Fuzhou 350108, China; baowenqiang@fjmu.edu.cn (W.B.); hongxinyu@fjmu.edu.cn (X.H.); lichutao@fjmu.edu.cn (C.L.); lujt@nxmu.edu.cn (J.L.); zhangdongcheng@stu.fjmu.edu.cn (D.Z.); 5 School of Basic Medical Sciences, Ningxia Medical University, Yinchuan 750000, China; 6 Fujian Key Laboratory of Tumor Microbiology, Department of Medical Microbiology, School of Basic Medical Sciences, Fujian Medical University, Fuzhou 350108, China

**Keywords:** humantenine, m6A modification, transcriptome, tight junction, actin cytoskeleton

## Abstract

Humantenine, an alkaloid isolated from the medicinal herb *Gelsemium elegans* (Gardner & Chapm.) Benth., has been reported to induce intestinal irritation, but the underlying toxicological mechanisms remain unclear. The object of the present study was to investigate the RNA N6-methyladenosine (m6A) modification and distinct mRNA transcriptome profiles in humantenine-treated HCT116 human colon cancer cells. High-throughput MeRIP-seq and mRNA-seq were performed, and bioinformatic analysis was performed to reveal the role of abnormal RNA m6A modification and mRNA expression in humantenine-induced intestinal cell toxicity. After humantenine treatment of HCT116 cells, 1401 genes were in the overlap of differentially m6A-modified mRNA and differentially expressed mRNA. The Kyoto Encyclopedia of Genes and Genomes and Gene Ontology annotation terms for actin cytoskeleton, tight junctions, and adherens junctions were enriched. A total of 11 kinds of RNA m6A methylation regulators were differentially expressed. The m6A methylation levels of target genes were disordered in the humantenine group. In conclusion, this study suggested that the HCT116 cell injury induced by humantenine was associated with the abnormal mRNA expression of m6A regulators, as well as disordered m6A methylation levels of target genes.

## 1. Introduction

*Gelsemium elegans* (Gardner & Chapm.) Benth. (*G. elegans*), an evergreen woody vine belonging to the family *Loganiaceae*, is a traditional medicinal herb with a drug use history of over 1800 years [1]. It is widely distributed in Southeast Asia, especially in Hunan, Guangdong, Fujian, and Yunnan provinces of China. In traditional Chinese medicine, *G. elegans* is used for the treatment of neuropathic pain, rheumatoid pain, swelling and skin ulcers, and also exerts anti-inflammatory, anti-tumor, anti-anxiety, and immunomodulatory effects [2]. However, *G. elegans* has been reported to be associated with severe gastrointestinal toxicity, such as vomiting, abdominal pain, diarrhea, or constipation, and even to induce symptoms of the nervous, circulatory, and respiratory systems, such as dizziness, drops in blood pressure, and respiratory paralysis [3]. To date, more than 2000 poisoning deaths have been reported in China [4]. According to the statistics from the Chinese Center for Disease Control and Prevention, the lethality rate of *G. elegans* was 15.8%, ranking second in the total vegetal food poisoning cases in China from 2004 to 2013 [5]. This lethality rate even reached 47.6% in Guangxi Province in 2015 to 2017, as reported by the foodborne disease incident monitoring and reporting system [6]. The severe side effects of *G. elegans* limit its clinical application.

There is an urgent need to reveal the toxicity mechanisms of *G. elegans* in order to allow the safe use of *G. elegans*, so the isolation and identification of toxic components is important. To date, nearly 200 components have been discovered in *G. elegans*, including alkaloids, iridoids, lipids, triterpene, steroids, and their glycosides, coumarins, flavones, lignans, phenolic acids, fructoses and their derivatives [7,8]. Among them, the alkaloids include 121 species, and indole alkaloids are the main biologically active components that exert pharmacological and toxicological effects. The most abundant indole alkaloids, from highest to lowest, are koumine, gelsevirine, gelsemine, humantenine, and gelsenicine. A previous study reported that the median lethal dose (LD_50_) of total alkaloids is 1.5 mg/kg.bw in female mice after intramuscular injection, and 1.2 mg/kg.bw in male rats after intraperitoneal injection [9]. The LD_50_ of koumine, gelsevirine *N*-oxide, gelsemine, and gelsenicine was also investigated in rodent experimental models, and the toxicity of humantenine has not been reported.

Previous studies suggested that the biological effects of alkaloids may be related to RNA epigeneticmodification [10,11]. In recent decades, about 160 RNA modifications have been reported, most of which are involved in the transcriptional and post-transcriptional regulation of genes [12]. The modification types include N1-methyladenosine (m1A) [13], 5-methylcytosine (m5C) [14], N6-methyladenosine (m6A), and 7-methylguanosine (m7G) [15], of which m6A is the most abundant and prevalent internal chemical modification in the mRNA of eukaryotes [16]. By means of liquid chromatography with tandem mass spectrometry, it has been reported that m6A has an abundance of 0.1–1.79% modifications per main base in mammals [17]. The m6A methylation modification sites are mostly located in stop codons and 3′ untranslated regions (3′UTRs) of mRNA [18]. In addition, the consensus motif RRACH (R = A/G, H = A/U/C) was found in m6A-modified RNA [19]. Regarding the biological function of RNA m6A modification, it plays an important role in epigenetic regulation and affects gene expression related to RNA splicing, translation, localization, and stability [20]. RNA m6A methylation is involved in a variety of biological processes, such as cell growth, development, metabolism, renewal, differentiation, immunity, and proliferation [21].

RNA m6A methylation is a dynamic and reversible process mainly regulated by m6A methyltransferases (writers), m6A methylation recognition proteins (readers), and m6A demethylases (erasers) together [22]. At the initial stage, the methyl group is transferred to the sixth nitrogen atom of adenine by the methyltransferase complex composed of METTL3, METTL14, and WTAP. The m6A-modified RNA can then be specifically recognized by YTH-domain family protein (YTHDF) and insulin-like growth factor 2 mRNA-binding protein (IGFBP) [23]. The readers play a vital role in the mRNA decay, storage, and stability [24]. At the same time, the m6A modification can be cleared by demethyltransferases, such as ALKBH5 and FTO.

The modification of RNA transcripts has been known since the 1970s. In recent years, the development of methylated RNA immunoprecipitation sequencing (MeRIP-seq) has made it possible to gain a thorough understanding of RNA m6A modification and decipher the corresponding biological functions [18]. In this study, we hypothesized that humantenine may alter RNA m6A modification in HCT116 human colon cancer cells and induce downstream biological adverse effects. High-throughput m6A MeRIP-seq and mRNA-seq were performed to reveal the potential molecular mechanisms of humantenine-induced HCT116 cell toxicity.

## 2. Materials and Methods

### 2.1. Chemical Reagent

**Figure 1:**
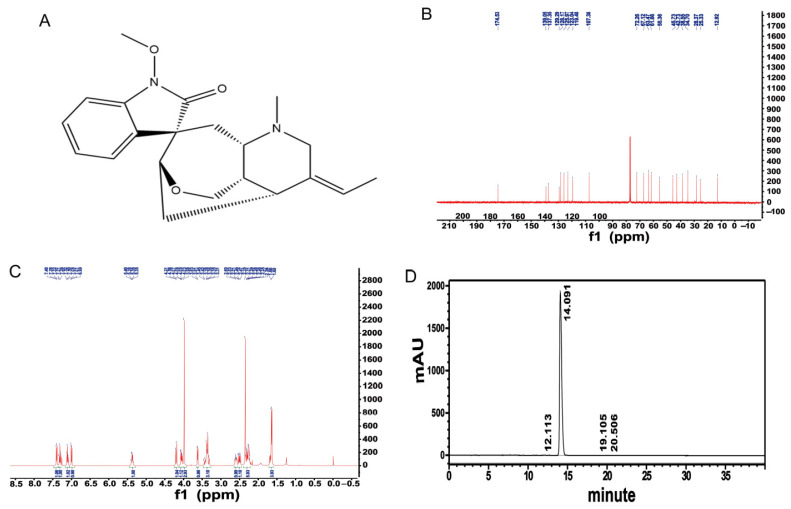
(**A**) The chemical structure of humantenine. The 400 MHz ^13^C (**B**) and ^1^H (**C**) NMR spectra of humantenine. (**D**) The purity of humantenine as determined by HPLC. Chromatographic conditions: Sinachrom ODS-BP (250 × 4.6 mm, 5 μm particle size); mobile phase of acetonitrile: water = 50: 50; column temperature: 35 °C; injection volume: 10 μL. The retention time of humantenine was 14.091 min, and the area percent of humantenine was 99.77%.

Humantenine (C_21_H_26_O_3_N_2_) was purchased from Must Bio-Technology Co., Ltd. (Chengdu, China). The chemical structure (Figure 1A) of humantenine was confirmed from the 400 MHz ^13^C and ^1^H nuclear magnetic resonance (NMR) spectra (Bruker, Rheinstetten, Germany) (Figure 1B,C). The purity of humantenine was 99.77% (Figure 1D), as determined by high-performance liquid chromatography (HPLC) (Shimadzu, Tokyo, Japan).

### 2.2. Cell Culture and Treatment

Human colon cancer cell line HCT116 was originally purchased from American Type Culture Collection. The cells were cultured in Dulbecco’s modified Eagle’s medium (DMEM, GE Healthcare Hyclone, Logan, UT, USA) supplemented with 10% fetal bovine serum (Gibco, New York, NY, USA), 100 μg/mL streptomycin sulfate and 100 U/mL penicillin G sodium salt, and then maintained in an incubator (Thermo Fisher, Langenselbold, Germany) with an atmosphere of 5% CO_2_ at 37 °C. Humantenine was dissolved in dimethyl sulfoxide (DMSO) to a concentration of 400 μM. The final working concentration of DMSO in cell culture experiments was 1%.

### 2.3. RNA Preparation

The cells were cultured in 9 cm dishes. After humanteinine treatment for 48 h, the cells were washed twice in PBS, and Trizol reagent (Invitrogen, Carlsbad, CA, USA) was added to lyse cells. Total RNA was extracted, and the concentration and integrity were measured via Qubit RNA HS Assay and Agilent 2100 Bioanalyzer (Santa Clara, CA, USA), respectively.

### 2.4. High-Throughput m6A MeRIP-seq and mRNA-seq

The m6A-modified RNA enrichment and sequencing was performed by E-GENE Tech Co., Ltd. (Shenzhen, China). Firstly, we used the Dynabeads™ mRNA Purification Kit (Invitrogen) to separate mRNA from the 44 μg total RNA. Subsequently, 1× RNA Fragmentation Buffer (Thermo Fisher, Langenselbold, Germany) was used at 70 °C for 5 min to turn purified mRNA into fragments. After purification, 5 μg of m6A antibody (Synaptic Systems, Goettingen, Germany) that had been incubated with 25 μL of protein A/G magnetic beads mixture (Thermo Fisher, Langenselbold, Germany) was taken out and used to enrich RNA. The incubation was then rotated at 4 °C for 4 h. After immunoprecipitation, the beads were washed three times with IP buffer (150 mM NaCl, 10 mM Tris-HCl, 0.1% IGEPAL CA-630 in nuclease-free H_2_O) for 10 min each time, rotating at 4 °C. Finally, m6A-modified mRNA fragments were extracted with Trizol reagent and purified using an RNA Clean & Concentrator Kit (Zymo Research, Orange, CA, USA). The purified m6A antibody-enriched RNA and some unenriched mRNA fragments were used as input to construct libraries with the VAHTS mRNA-seq V3 Library Prep Kit for Illumina (Vazyme, Nanjing, China) according to the manufacturer’s protocol. The established libraries were analyzed using an Agilent 2100 Bioanalyzer, quantified by real-time PCR, and then sequenced on the NovaSeq 6000 platform (Illumina, San Diego, CA, USA). Three independent samples were used in each group of m6A MeRIP-seq and mRNA-seq.

### 2.5. Sequencing Data Analysis

The raw data were processed by Trim Galore (Cambridge, UK). The sequencing results were aligned with the human genome reference hg19 using Hisat2 [25], the RNA expression level was analyzed using StringTie (Baltimore, MD, USA) [26], and differential expression was calculated using DEseq [27]. The exomepeak2 (Suzhou, China) [28,29] was applied for m6A peak calling and the detection of differential methylation. In exomepeak2, the Poisson Generalized Linear Model was used to estimate the methylation level and detect differential methylation regions. The exomePeak2′s estimation of sequencing depth size factors was on non-methylation background regions. The consensus of m6A motif sequences was identified by STREME [30]. The STREME algorithm integrated the position weight matrix Markov model to report a useful estimate of the statistical significance of each motif it discovered. The Kyoto Encyclopedia of Genes and Genomes (KEGG) and Gene Ontology (GO) annotations were done by KOBAS (Beijing, China) [31] and DAVID (Frederick, MD, USA) [32], respectively. The distribution of epitranscriptome profiles was visualized by MetaTX (Suzhou, China) [33]. The m6A conservation and disease association were obtained from ConsRM (Suzhou, China) [34] and RMDisease (Suzhou, China) [35]; the substrates of m6A regulators identified by CLIP techniques were downloaded from starBase v2.0 (Guangzhou, China) [36]. The m6A patterns of gene methylated sites were visualized using Integrative Genomics Viewer (IGV) software. Protein–protein interaction (PPI) analysis was performed using STRING and visualized using Cytoscape.

### 2.6. Molecular Docking

To explore whether humantenine could interact with differentially expressed regulators of m6A modification, the SYBYL-X 2.0 software (Tripos, St Louis, MO, USA) was applied to perform molecular docking. The 3D structures of proteins were retrieved from the Protein Data Bank (PDB). The protein structures were prepared using SYBYL-X 2.0 to remove water molecules and heteroatoms, add hydrogen atoms, and repair side chains [37]. The 2D structure of humantenine was downloaded from the PubChem compound database and was imported into Chem3D (Waltham, MA, USA) to generate 3D structures according to the energy minimization principle. After being stored in mol2 format, the 3D structure of humantenine was set to the lowest energy conformation to mimic the stable molecular conformation in the natural system [38]. Proteins were docked in Surflex-Dock GeomX mode through the semi-flexible docking method. The total score, which is a comprehensive evaluation of hydrophobic complementarity, polar complementarity, solvation terms, and entropic terms, was deemed a stable interaction when the value was higher than 5 [39].

## 3. Results

### 3.1. Transcriptome-Wide Detection of m6A Modification after Humantenine Treatment of HCT116 Cells

**Figure 2:**
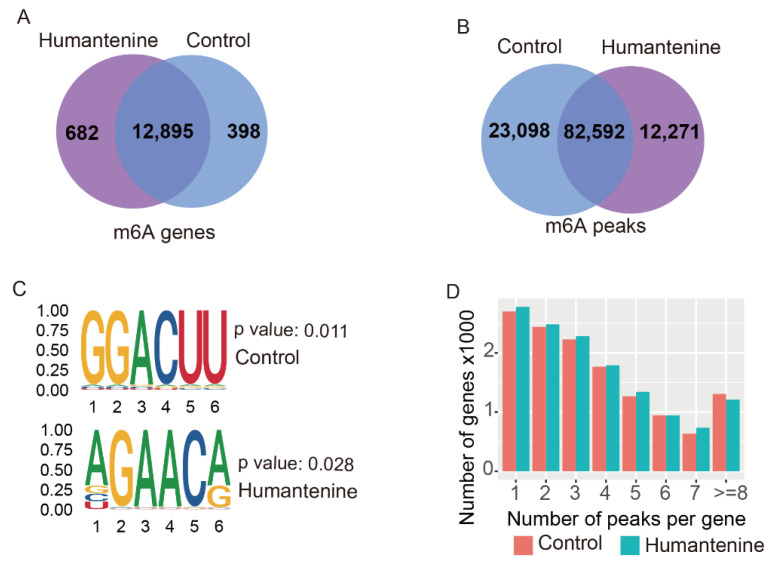
The m6A modification patterns in humantenine and control groups. (**A**) Venn diagram showing overlap of the m6A genes from both groups. (**B**) Venn diagram showing overlap of the m6A peaks from both groups. (**C**) The motifs for m6A peak regions based on STREME. (**D**) The distribution of m6A methylation peaks per gene.

To explore the mechanism of action of humantenine in HCT116 human colon cancer cells, MeRIP-seq analysis and RNA-seq were performed. In the humantenine group, 94,863 m6A peaks containing transcripts of 13,577 genes were identified by the R package exomePeak. Similarly, 105,690 m6A peaks were identified in the control group, representing transcripts of 13,293 genes. In addition, 82,592 peaks corresponding to 12,895 genes were found at the intersection of the humantenine and control groups (Figure 2A,B). We then used STREME to determine whether the m6A consensus sequence of RRACH (where R represents purine, A is m6A, and H is a non-guanine base) was reported in the detection of m6A (Figure 2C). The result showed the existence of some motifs, including the classical consensus sequence in the control and humantenine groups. Notably, the majority of genes had 1–3 m6A methylation peaks, whereas a relatively small number of genes contained four or more m6A methylation peaks in both humantenine and control groups (Figure 2D).

### 3.2. Distribution of m6A Modification in the Transcriptome

**Figure 3:**
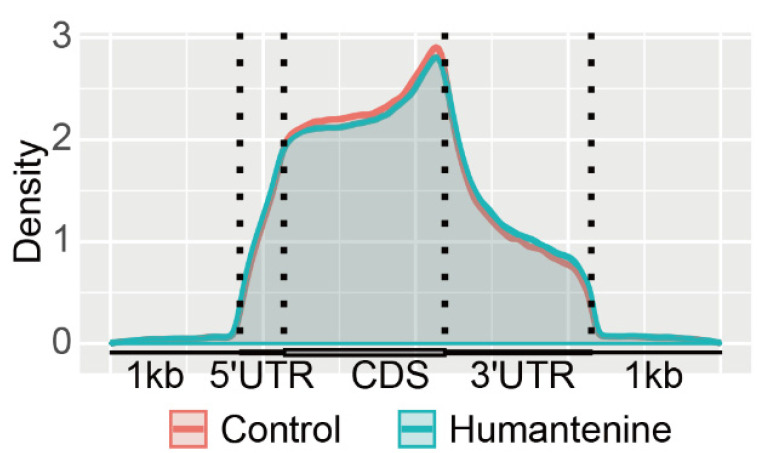
Density of m6A methylation peaks in mRNA transcripts.

The distributions of m6A methylation in the whole transcriptomes of the humantenine and control groups were analyzed. The result showed that m6A modification tended to enrich in five transcript segments: 5′untranslated region (5′UTR), the start codon segment, coding sequence (CDS), the stop codon segment, and 3′UTR. The m6A peak density increased rapidly between the 5′UTR and the start codon and was relatively gentle in the CDS region. The highest density region was near the stop codon. In the 3′UTR region, the density of the m6A peaks decreased rapidly (Figure 3).

### 3.3. Differentially Methylated Genes and Differentially Expressed Genes

**Figure 4:**
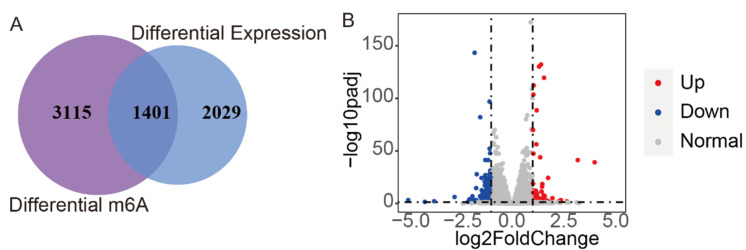
(**A**) Differential m6A modified mRNA and differential mRNA expression in humantenine and control groups. (**B**) The volcano plot of differentially expressed mRNAs between both groups.

Setting the statistical standard as *p* ≤ 0.05, a total of 4516 differential m6A-modified mRNAs were found through m6A-seq data, and 3430 mRNAs were determined to be differentially expressed between humantenine and control groups after the analysis of RNA-seq data. In addition, 1401 genes were consistently observed at the overlap of differentially m6A-modified mRNA and differentially expressed mRNA (Figure 4A). When the statistical standard was set as fold change ≥ 1 and *p* ≤ 0.05, 681 differentially expressed mRNAs were screened out, of which 248 were up-regulated and 433 were down-regulated (Figure 4B).

### 3.4. KEGG and GO Annotation of the Overlap of Differentially m6A-Modified mRNA and Differentially Expressed mRNA

**Figure 5:**
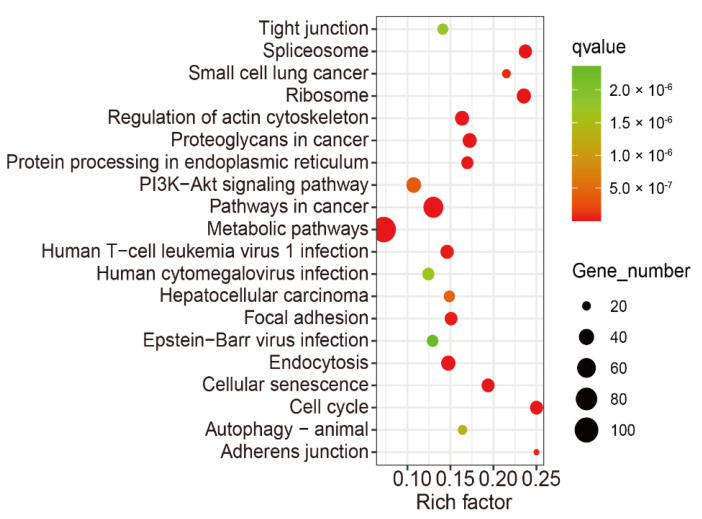
The top 20 KEGG analysis enriched pathways of the 1401 overlapped genes of differentially m6A modified mRNA and differentially expressed mRNA.

**Figure 6:**
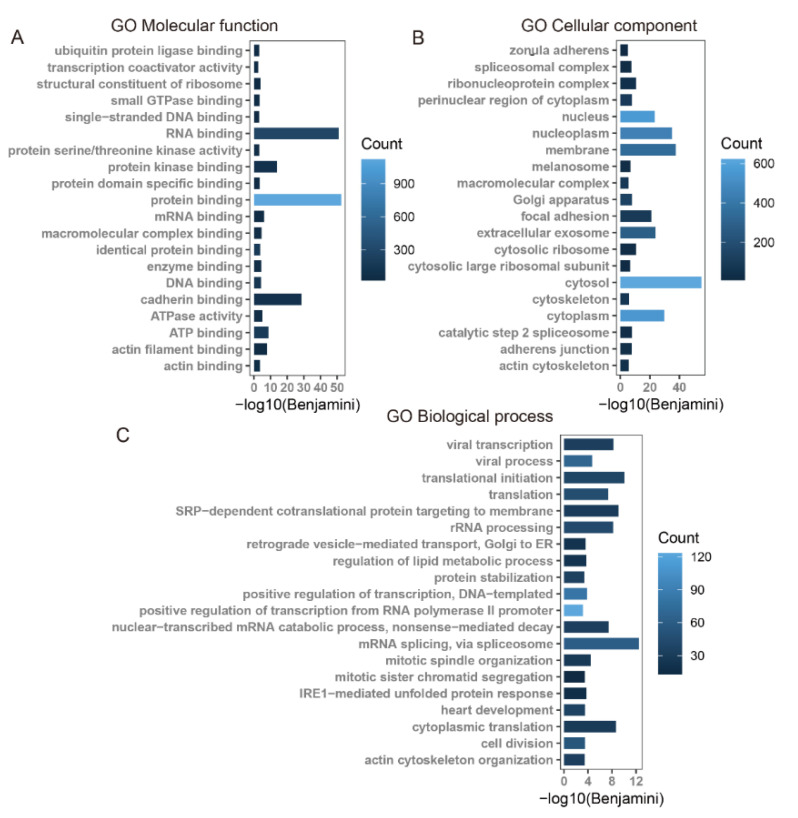
GO functional annotation of 1401 overlapped genes of differentially m6A-modified mRNAs and differentially expressed mRNAs. (**A**) GO molecular function. (**B**) GO cellular component. (**C**) GO biological process.

Using KEGG pathway and GO enrichment analysis on the DAVID web server, the 1401 overlapping genes of differentially m6A-modified mRNA and differentially expressed mRNAs were associated with significant pathways and biological functions. The KEGG pathway analysis revealed that these genes were mainly involved in tight junction, regulation of actin cytoskeleton, metabolic pathways, focal adhesion, and adherens junction pathways, and so on (Figure 5). The GO enrichment analysis was classified into three functional types: molecular function (MF), cellular component (CC), and biological process (BP). The MF terms included actin filament binding and actin binding. The CC terms included zonula adherens, cytoskeleton, adherens junction, and actin cytoskeleton. The BP terms included actin cytoskeleton organization (Figure 6).

### 3.5. The m6A Conservation and Disease Association

**Figure 7:**
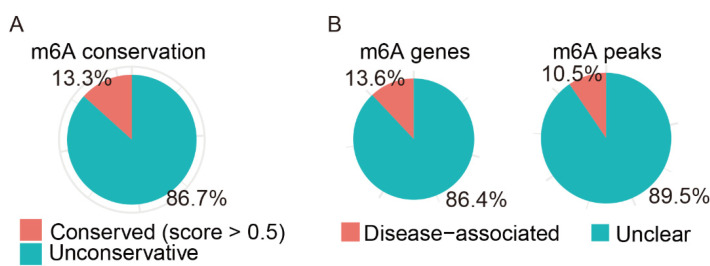
Analysis of m6A conservation and disease association. (**A**) The conserved and un-conserved m6A sites in differentially m6A-modified mRNA. (**B**) The disease-associated m6A genes and peaks in differentially m6A-modified mRNA.

As shown in Figure 7A, based on the analysis of ConsRM, 86.7% of m6A-modified sites were nonconservative in the 1401 overlapping genes of differentially m6A-modified mRNAs and differentially expressed mRNAs. As shown in Figure 7B, the results of the RMDisease analysis showed that 13.6% of m6A genes and 10.5% of m6A peaks were associated with diseases.

### 3.6. Gene Expression of Tight Junctions, Adherens Junctions, and Regulation of Actin Cytoskeleton, and Their Potential Regulators

According to the RNA-seq data in HCT116 cells, after treatment with humantenine, the mRNA expression levels of genes associated with tight junctions (*CLDN4*, *CDK4*, *TJP3*, *MAGI1*), adherens junctions (*ACTN1*, *NECTIN2*, *WASF1*), and regulation of actin cytoskeleton (*MYH9*, *IQGAP3*, *DIAPH3*, *WASF1*, *ARPC5L*, *DOCK1*) were significantly differentially expressed (Table 1). The potential regulators, including parts of writers, readers, and erasers, were identified using CLIP techniques. The results showed that the reader IGF2BP3 was involved in the regulation of all these genes.

### 3.7. Potential RNA m6A Regulators of Differentially Methylated Genes

In order to identify the potential regulators of RNA m6A methylation, we analyzed the mRNA expression levels of 22 RNA m6A methylation writers, readers, and erasers. As shown in Table 2, four writers, six readers, and one eraser were significantly differentially expressed (padj ≤ 0.05). Among them, writers *RBM15* and *METTL3*, reader *YTHDF3*, and eraser *ALKBH5* were up-regulated; writers *RBM15B* and *ZC3H13*, and readers *HNRNPA2B1*, *YTHDF2*, *YTHDC2*, *IGF2BP3*, and *YTHDC1* were down-regulated.

**Figure 8:**
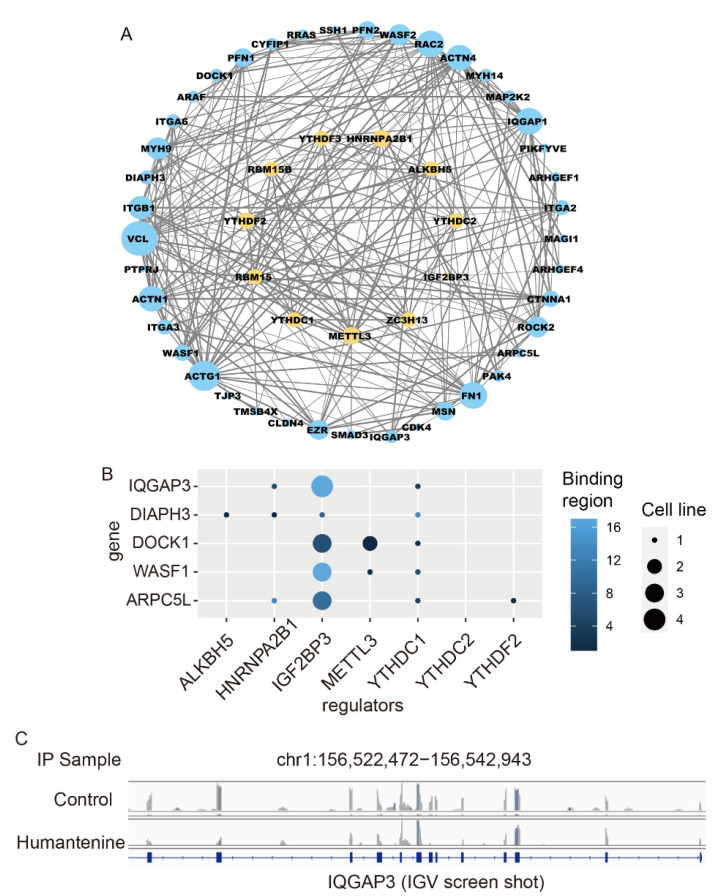
(**A**) The protein-protein interactions between m6A regulators (yellow circle) and actin cytoskeleton, tight junction, and adherens junction factors (blue circle). (**B**) The effect of m6A regulators on the differentially expressed genes of actin cytoskeleton. (**C**) The level of m6A on IQGAP3 mRNA transcripts observed by IGV.

Since the GO and KEGG terms included tight junctions, adherens junctions, and actin cytoskeleton, as shown in Figure 5 and Figure 6, the genes enriched in these terms were collected. We then analyzed the regulatory relationship between these genes and 11 differentially expressed RNA m6A methylation regulators in a PPI network (Figure 8A). The degrees of the 11 regulators ALKBH5, HNRNPA2B1, YTHDF2, YTHDC2, IGF2BP3, RBM15B, YTHDF3, YTHDC1, RBM15, METTL3, and ZC3H13 were 9, 11, 10, 9, 3, 9, 9, 9, 9, 11, and 9, respectively.

To explore the substrates of RNA m6A methylation regulators, the writer METTL3, readers HNRNPA2B1, IGF2BP3, YTHDC1, YTHDC2, and YTHDF2, and eraser ALKBH5 were selected, as well as the actin cytoskeleton genes *IQGAP3*, *DIAPH3*, *DOCK1*, *WASF1*, and *ARPC5L*. The result showed that insulin-like growth factor 2 mRNA-binding protein 3 (IGF2BP3) was involved in the regulation of all five of these actin cytoskeleton genes (Figure 8B). As indicated by the IGV display of *IGF2BP3*, the m6A methylation level on mRNA transcripts was decreased after humantenine treatment (Figure 8C).

### 3.8. Molecular Interactions of Humantenine with Differently Expressed Regulators of RNA m6A Modification

**Figure 9:**
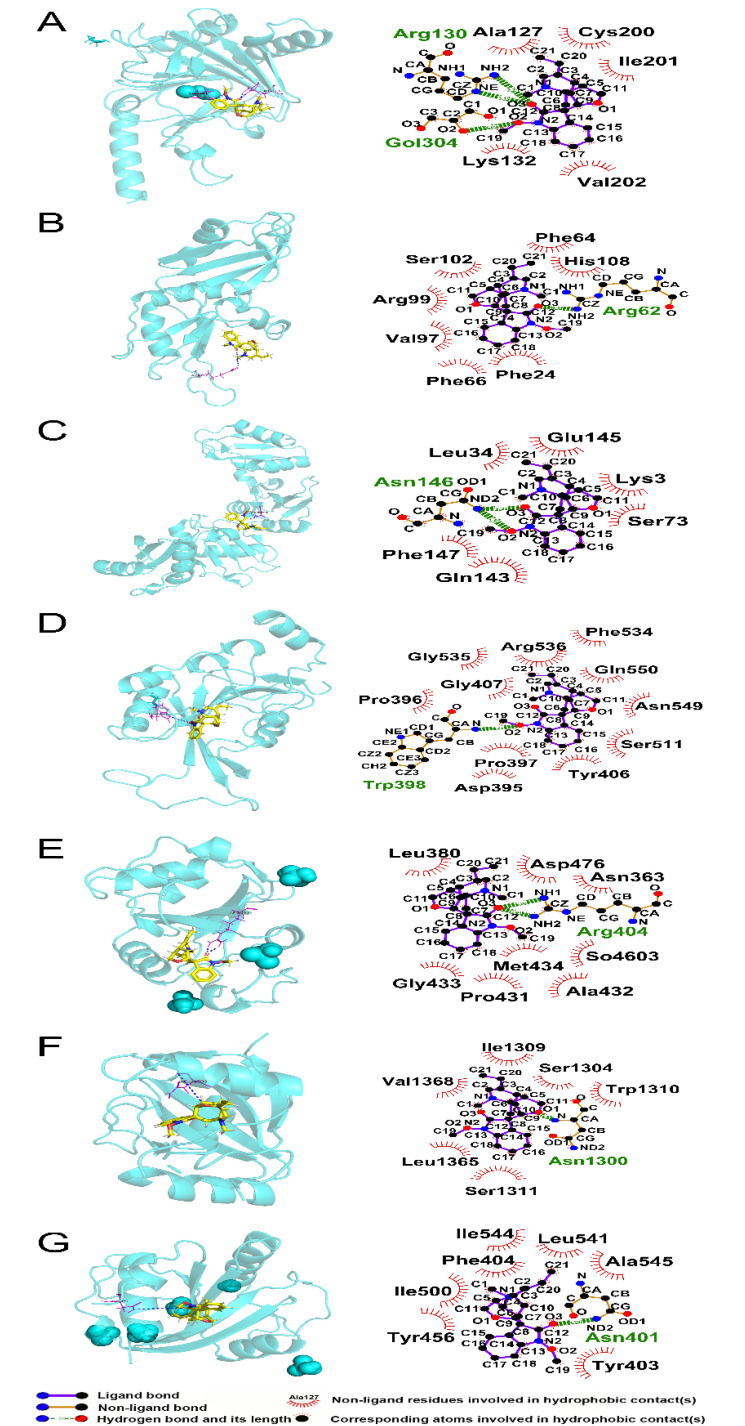
Molecular interactions between humantenine and differently expressed regulators of m6A modification. Both 3D and 2D interaction models were presented. (**A**) ALKBH5, (**B**) HNRNPA2B1, (**C**) IGF2BP3, (**D**) METTL3, (**E**) YTHDC1, (**F**) YTHDC2 and (**G**) YTHDF2.

We evaluated the molecular interactions of humantenine with differentially expressed regulators of m6A modification, including ALKBH5, HNRNPA2B1, IGF2BP3, METTL3, YTHDC1, YTHDC2, and YTHDF2. The results are shown in Figure 9 and Table 3. All the total scores were over 5, which suggested stabilized interactions between humantenine and these regulators.

Theoretically, humantenine bound ALKBH5 via the formation of two hydrogen bonds at Arg 130 and one hydrogen bond at Gol304, and five hydrophobic contacts with Ala127, Lys132, Cys200, Ile201, and Val202 (Figure 9A). Humantenine bound HNRNPA2B1 via the formation of one hydrogen bond at Arg62, and seven hydrophobic contacts with Phe24, Phe64, Phe66, Val97, Arg99, Ser102, and His108 (Figure 9B). Humantenine bound IGF2BP3 via the formation of two hydrogen bonds at Asn146, and six hydrophobic contacts with Lys3, Leu34, Ser73, Gln143, Glu145, and Phe147 (Figure 9C). Humantenine bound METTL3 via the formation of one hydrogen bond at Trp398, and eleven hydrophobic contacts with Asp395, Pro396, Pro397, Tyr406, Gly407, Ser511, Phe534, Gly535, Arg536, Asn549, and Gln550 (Figure 9D). Humantenine bound YTHDC1 via the formation of two hydrogen bonds at Arg404 and eight hydrophobic contacts with Asn363, Leu380, Pro431, Ala432, Gly433, Met434, Asp476, and So4603 (Figure 9E). Humantenine bound YTHDC2 via the formation of one hydrogen bond at Asn1300 and six hydrophobic contacts with Ser1304, Ile1309, Trp1310, Ser1311, Leu1365, and Val1368 (Figure 9F). Humantenine bound YTHDF2 via the formation of one hydrogen bond at Asn401 and seven hydrophobic contacts with Tyr403, Phe404, Tyr456, Ile500, Leu541, Ile544, and Ala545 (Figure 9G).

## 4. Discussion

The traditional medicine *G. elegans* exerts excellent pharmacological therapeutic effects in the treatment of a variety of clinical symptoms, especially neuralgia and inflammation. On the other hand, the severe side effects in multiple systems limit its wider application. Therefore, it is in urgent need to reveal the molecular mechanism of *G. elegans*-induced intestinal irritation, for the clinic drug safety. In the present study, the indole alkaloid humantenine was used in human intestinal cells to analyze the potential epigenetic alteration of RNA m6A modification, and the mRNA transcriptional profile, based on high-throughput m6A MeRIP-seq and mRNA-seq.

Through analysis of m6A MeRIP-seq data, the motif sequence was successfully identified with statistical significance in the region around the m6A peak of the control group and humantenine group. The consensus motif sequence RRACH was observed in the m6A motif region, as proven previously [40,41]. As to the distribution of m6A modification peaks, most genes had 1~3 peaks, and parts of genes contained more peaks, which was consistent with the study by Chen et al. [42]. When the genes were divided into 5′UTR, start codon, CDS, stop codon, and 3′UTR, most m6A modification peaks were in the CDS region, and the highest value was around the stop codon, as frequently reported in the past [40,43]. A higher number of m6A modification sites at the stop codon may be associated with mRNA stability and translation [44].

In the present study, humantenine treatment induced 86.7% RNA m6A modification in un-conserved sites, and 13.6% of genes were associated with diseases, suggesting that this xenobiotic compound might cause intestinal diseases. To explore the biological function change induced by RNA m6A modification, GO and KEGG pathway enrichment analysis was performed. After humantenine treatment in HCT116, a total of 1041 genes was reported in the overlap of the differentially m6A-modified mRNA and differentially expressed mRNA. The top 20 KEGG terms included tight junction, regulation of actin cytoskeleton, and adherens junction. In the GO terms, cadherin binding, actin filament binding, and actin binding were enriched in MF; zonula adherens, focal adhesion, cytoskeleton, adherens junction, and actin cytoskeleton were enriched in CC; actin cytoskeleton organization was enriched in BP. In clinic, diarrhea is the main intestinal side effect of *G. elegans*, manifested by increased intestinal permeability, which is regulated by intestinal intercellular junction and actin cytoskeleton. The intercellular junction mainly comprises tight junctions and adherens junctions, and acts as the mechanical barrier in defense against exogenous toxic compounds [45]. It also regulates the paracellular transport pathway to maintain intestinal cell integrity [46,47]. Kumar et al. found that *Cryptosporidium parvum* disrupted intestinal barrier function by downregulating the expression of tight junctions and adherens junctions, resulting in diarrhea [48]. In this study, expression levels of multiple mRNAs involved in tight junctions, adherens junctions, and regulation of actin cytoskeleton were significantly differentially expressed in the humantenine-treated group. These genes encoded proteins of claudin 4, zonula occludens protein 3, actinin α 1, nectin cell adhesion molecule 2, Wiskott–Aldrich syndrome protein family member 1, myosin heavy chain 9, IQ motif containing GTPase activating protein 3 (IQGAP3), diaphanous related formin 3, wasp family member 1, actin related protein 2/3 complex subunit 5, dedicator of cytokinesis 1, and so on. If the junction proteins and actin cytoskeleton were destroyed by humantenine, the paracellular pathway transport would be in disorder, characterized by abnormal osmotic pressure and permeability in intestinal cells [49,50].

RNA m6A modifications were mediated by three specific categories of proteins, namely m6A methyltransferases, m6A demethylases, and m6A-biding proteins. The biological effects of m6A methylation modifications depend on the recognition and binding of m6A-binding proteins [51]. Using the computational prediction method, humantenine was combined with the m6A regulators with total scores higher than 5. Therefore, the present study further tried to identify the m6A regulators of the differentially expressed genes related to intercellular junctions and actin cytoskeleton. A total of 11 m6A regulators were differentially expressed, of which IGF2BP3, an m6A reader exerting the main function of enhancing the stability of mRNA [52], was regarded as the core factor in the regulation of mRNA translation, decay, and storage. For example, IQGAP3 is a Ras GTPase-activating-like protein that belongs to the family of scaffolding proteins and regulates cell adhesion by mediating the actin cytoskeleton [53]. The m6A methylated mRNA of *IQGAP3* can be recognized by IGF2BP3, thus maintaining a stable state in nuclear and cytoplasmic conditions. As downstream biological effects, the translation and storage of *IQGAP3* mRNA were promoted and the decay of *IQGAP3* mRNA was inhibited. In this study, humantenine inhibited the mRNA m6A methylation level of *IQGAP3*, making it difficult for IGF2BP3 to recognize and bind *IQGAP3*. As a result, the mRNA stability and expression level of *IQGAP3* decreased, which would cause the actin cytoskeleton to collapse and thus enhance intestinal permeability.

## 5. Conclusions

**Figure 10:**
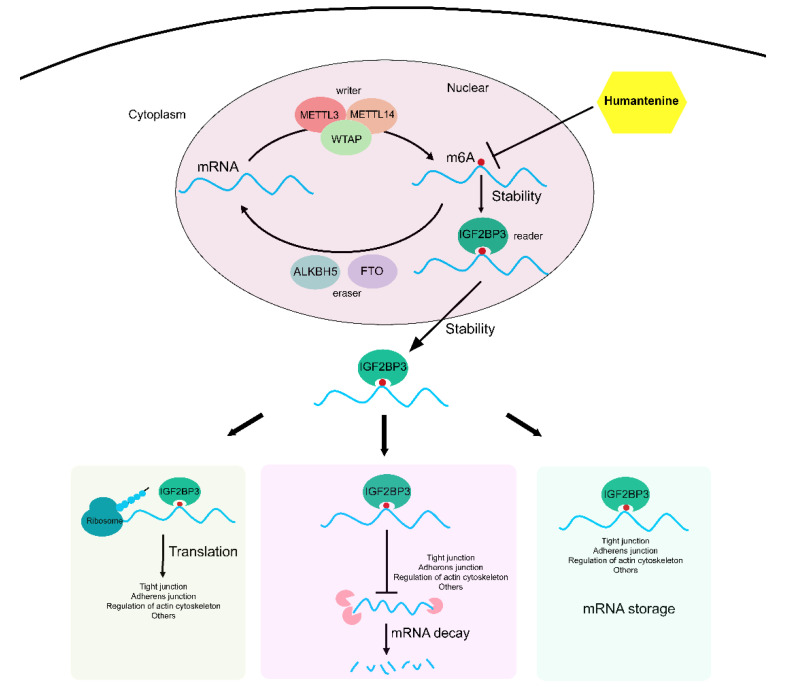
Humantenine induced the abnormal m6A methylation of genes involved in tight junctions, adherens junctions, and regulation of actin cytoskeleton. It consequently disrupted mRNA translation, storage, and decay.

In summary, the present study investigated the mRNA m6A modification and expression profiles of HCT116 human colon cancer cells treated with humantenine. The abnormal mRNA expression of m6A regulators, together with disordered m6A methylation levels of target genes, led to the disruption of mRNA stability, translation, storage, and decay (Figure 10). This study has therefore revealed a possible mechanism for the increased intestinal permeability induced by humantenine.

## Figures and Tables

**Table 1:** The mRNA expression levels of tight junction, adherens junction, regulation of actin cytoskeleton, and their potential regulators.

	Gene	log2FoldChange	padj	Writer	Reader					Eraser
				METTL3	HNRNPA2B1	IGF2BP3	YTHDC1	YTHDC2	YTHDF2	ALKBH5
Tight junction	*CLDN4*	0.5708	8.04 × 10^−32^	N	N	Y	N	N	N	N
	*CDK4*	0.4725	4.43 × 10^−26^	Y	Y	Y	Y	N	N	N
	*TJP3*	−0.9036	1.84 × 10^−67^	N	N	Y	N	N	N	N
	*MAGI1*	−0.4761	6.83 × 10^−16^	Y	Y	Y	Y	N	N	N
Adherens junction	*ACTN1*	0.2529	3.31 × 10^−11^	Y	Y	Y	Y	N	N	N
	*NECTIN2*	0.2513	1.58 × 10^−5^	N	Y	Y	Y	N	N	N
	*WASF1*	−0.4224	9.38 × 10^−5^	Y	N	Y	Y	N	N	N
Regulation of actin cytoskeleton	*MYH9*	0.3207	2.97 × 10^−22^	Y	Y	Y	Y	N	N	N
	*IQGAP3*	−0.8892	3.82 × 10^−16^	N	Y	Y	Y	N	N	N
	*DIAPH3*	−0.6702	2.45 × 10^−9^	N	Y	Y	Y	N	N	Y
	*WASF1*	−0.4224	9.38 × 10^−5^	Y	N	Y	Y	N	N	N
	*ARPC5L*	−0.3197	1.42 × 10^−4^	N	Y	Y	Y	N	Y	N
	*DOCK1*	−0.3172	8.64 × 10^−6^	Y	N	Y	Y	N	N	N

Y means the mRNA was the substrate of the RNA m6A regulators, and N means not.

**Table 2:** The mRNA expression levels of m6A regulators in humantenine-treated HCT116 cells.

Gene	Regulation	Base Mean	log2FoldChange	padj
*ALKBH5*	eraser	5355.58	0.5337	2.43 × 10^−18^
*HNRNPA2B1*	reader	31,317.46	−0.2827	3.61 × 10^−13^
*YTHDF2*	reader	5827.43	−0.3176	1.44 × 10^−7^
*YTHDC2*	reader	1996.91	−0.3977	9.40 × 10^−5^
*IGF2BP3*	reader	7360.70	−0.1831	5.89 × 10^−4^
*RBM15B*	writer	4931.66	−0.2692	5.93 × 10^−4^
*YTHDF3*	reader	4575.69	0.2471	2.44 × 10^−3^
*YTHDC1*	reader	4745.43	−0.1911	3.73 × 10^−3^
*RBM15*	writer	1909.31	0.3036	6.91 × 10^−3^
*METTL3*	writer	1801.37	0.2508	2.14 × 10^−2^
*ZC3H13*	writer	8932.93	−0.1169	2.65 × 10^−2^
*VIRMA*	writer	6469.09	0.1091	1.41 × 10^−1^
*FTO*	eraser	2646.90	0.1141	2.69 × 10^−1^
*IGF2BP1*	reader	65.30	0.5237	3.67 × 10^−1^
*YTHDF1*	reader	5181.16	0.0458	5.94 × 10^−1^
*HNRNPC*	reader	24,691.72	−0.0436	6.68 × 10^−1^
*METTL14*	writer	2711.17	0.0432	7.66 × 10^−1^
*METTL5*	writer	1640.50	0.0410	7.80 × 10^−1^
*CBLL1*	writer	2958.85	−0.0289	8.24 × 10^−1^
*IGF2BP2*	reader	8415.70	−0.0156	8.55 × 10^−1^
*FMR1*	reader	2456.26	−0.0059	9.70 × 10^−1^
*WTAP*	writer	2760.03	0.0004	9.98 × 10^−1^

**Table 3:** Molecular interactions between humantenine and differently expressed regulators of m6A modification.

Protein	PDB ID	Total Score	Crach	Polar	H-Bond Number	Residues Involved in H-Bond Formation	Hydrophobic Contacts Number	Residues Involved in Hydrophobic Contacts
ALKBH5	4NJ4	6.7567	−0.8591	1.9259	3	Arg130 (2 hydrogen bonds), Gol304	5	Ala127, Lys132, Cys200, Ile201, Val202
HNRNPA2B1	5HO4	5.1469	−0.5213	0.9835	1	Arg62	7	Phe24, Phe64, Phe66, Val97, Arg99, Ser102, His108
IGF2BP3	6FQ1	6.5409	−0.9417	1.2075	2	Asn146 (2 hydrogen bonds)	6	Lys3, Leu34, Ser73, Gln143, Glu145, Phe147
METTL3	5IL2	6.09	−1.0199	0.001	1	Trp398	11	Asp395, Pro396, Pro397, Tyr406, Gly407, Ser511, Phe534, Gly535, Arg536, Asn549, Gln550
YTHDC1	6ZCN	5.3546	−0.3608	2.0979	2	Arg404 (2 hydrogen bonds)	8	Asn363, Leu380, Pro431, Ala432, Gly433, Met434, Asp476, So4603
YTHDC2	6K6U	6.2361	−0.9342	0	1	Asn1300	6	Ser1304, Ile1309, Trp1310, Ser1311, Leu1365, Val1368
YTHDF2	7R5F	6.2342	−0.7086	0	1	Asn401	7	Tyr403, Phe404, Tyr456, Ile500, Leu541, Ile544, Ala545

## Data Availability

All relevant data is provided in the manuscript. Please contact at zhuan@fjmu.edu.cn for any raw data files and further analysis.

## Abbreviations

3′UTR	3′ untranslated regions
5′UTR	5′ untranslated regions
CDS	coding sequence (CDS)
*G. elegans*	*Gelsemium elegans* (Gardner & Chapm.) Benth.
GO	Gene Ontology
HPLC	high-performance liquid chromatography
IGF2BP3	insulin-like growth factor 2 mRNA-binding protein 3
IGV	Integrative Genomics Viewer
IQGAP3	IQ motif containing GTPase activating protein 3
KEGG	Kyoto Encyclopedia of Genes and Genomes
m6A	N6-methyladenosine
MeRIP-seq	methylated RNA immunoprecipitation sequencing
NMR	nuclear magnetic resonance
PPI	protein–protein interaction
